# The impact of the COVID-19 pandemic on vaccinations in United States primary care practices

**DOI:** 10.1371/journal.pone.0325934

**Published:** 2025-06-10

**Authors:** Ömer Ataç, Lars E. Peterson, Teresa M. Waters

**Affiliations:** 1 Department of Health Management and Policy, College of Public Health, University of Kentucky, Lexington, Kentucky, United States of America; 2 Department of Public Health, International School of Medicine, Istanbul Medipol University, Istanbul, Türkiye; 3 American Board of Family Medicine, Lexington, Kentucky, United States of America; 4 Department of Family and Community Medicine, College of Medicine, University of Kentucky, Lexington, Kentucky, United States of America; 5 School of Public Health, Augusta University, Augusta, Georgia, United States of America; Dilla University, ETHIOPIA

## Abstract

**Background:**

The COVID-19 pandemic caused substantial burdens for patients and our healthcare delivery system. Many patients delayed seeking care for essential medical needs, and providers struggled to deliver services. This study aimed to examine the impact of the COVID-19 pandemic on administration of non-COVID-19 vaccinations through primary care practices, using a nationwide sample.

**Methods:**

In this retrospective cross-sectional study, clinical records from the American Board of Family Medicine’s (ABFM) PRIME Registry for March 15, 2019 through March 14, 2022, were used to calculate monthly visit and vaccination rates among child-adolescents and adults, comparing Pre-Pandemic Year and Pandemic Years 1 and 2. Logistic regression was used to examine the association of patient characteristics with vaccination likelihood.

**Results:**

The number of vaccinated individuals decreased by 9.6% among children-adolescents and 4.2% among adults in Pandemic Year 1. In the Pandemic Year 2, the decline had worsened as 19.4% for children-adolescents and 14.2% for adults compared to the Pre-Pandemic Year. Despite a partial rebound in visit rates, vaccination rates did not fully recover. Except few months, differences in vaccination rates were worse than those in visit rates throughout the rest of the pandemic. Females, rural residents and individuals living in areas with higher social risk had the lowest likelihood of vaccination and this gap increased during the pandemic.

**Conclusions:**

The pandemic was associated with a decline in non-COVID-19 vaccinations that persisted through the second year of the pandemic. This decline raises concerns that delayed or missed vaccinations may trigger outbreaks of preventable diseases and the resurgence of diseases that were previously under control.

## Introduction

Vaccination has long been recognized as a paramount public health intervention, playing a crucial role in preventing and controlling infectious diseases across all age groups—from children to adults [1,2]. Vaccines significantly contribute to population health by reducing the incidence of preventable diseases, lowering morbidity and mortality rates, and fostering overall health status [3]. Interruptions in vaccination programs can lead to the accumulation of susceptible individuals within the general population, thereby heightening the risk of the resurgence of diseases that were previously under control or eliminated [1].

Primary care plays a pivotal role in vaccination services, encompassing the administration of vaccines and the provision of patient counseling regarding the risks and benefits of vaccination for various indications [4]. It is also integral to the delivery of preventive services, and patients with access to primary care are more likely to stay informed about a spectrum of preventive measures [5]. The COVID-19 pandemic disrupted primary care and various preventive services, including vaccinations [6–10]. Disparities in the receipt of preventive health care services have long been noted, with disadvantaged groups often experiencing lower vaccination rates [7,11]. Current evidence indicates that disruptions caused by COVID-19 have further widened these disparities, particularly affecting vulnerable groups [12–15]. Factors such as socioeconomic status, race/ethnicity, place of residence, educational attainment, and insurance status are associated with reduced vaccination rates [2,3].

Numerous studies have investigated changes in vaccination rates during the pandemic, revealing a significant decline of up to 94% compared to pre-pandemic rates [11,14–23]. Although these changes varied over time, the most pronounced decrease occurred in the first month. While some studies have indicated subsequent improvements in vaccination rates, this recovery has been inconsistent. Notably, most research has focused exclusively on pediatric populations in specific regions [16–18,22]. There is limited evidence assessing the effects of the pandemic on vaccination across all age groups in primary care settings. In a nationwide study included commercially insured individuals of all age groups, findings revealed a decrease in vaccination rates across all ages [11].

While most prior research has focused on pediatric immunizations, there is a need to examine vaccination trends across the life course. Adolescents and adults are also recommended to receive routine immunizations, including tetanus, diphtheria, and acellular pertussis (Tdap), human papillomavirus (HPV), meningococcal vaccines, influenza, and pneumococcal vaccines. Missed or delayed administration of these vaccines during the pandemic may increase susceptibility to preventable diseases, including outbreaks in older populations with comorbidities.

While current evidence has shown declines in vaccination rates during the COVID-19 pandemic, most studies focus on pediatric populations or specific regions. Our objective was to assess the impact of the COVID-19 pandemic on non-COVID-19 vaccination services delivered through primary care for various subpopulations, including children, adolescents, and adults.

## Methods

### Data and study design

We conducted a retrospective cross-sectional study to evaluate changes in the receipt of vaccination in primary care during the COVID-19 pandemic. We used the clinical records from the American Board of Family Medicine’s (ABFM) PRIME Registry. PRIME is a certified outpatient clinical quality data registry that collects electronic health record (EHR) data from participating primary care practices using automated feeds. The PRIME registry, developed by the ABFM, was established to facilitate quality reporting and improvement in primary care. Currently, PRIME houses data from over 3,000 primary care clinicians who are caring for patients in 1,250 practices across 50 states. This database includes information pertaining to the care of 6.5 million patients across 70 million visits. Although participation in PRIME is voluntary, the registry captures a wide range of patient encounters from diverse geographic areas, including urban, suburban, and rural settings. We accessed de-identified retrospective medical data on 03/02/2023. No identifying information was available to the authors at any point during or after the data collection process.

To assess the potential impact of the COVID-19 pandemic, we defined our study period from March 15, 2019, to March 14, 2022. This period was segmented into three distinct time windows: The Pre-Pandemic Year (March 15, 2019, to March 14, 2020), Pandemic Year 1 (Y1) (March 15, 2020, to March 14, 2021), and Pandemic Year 2 (Y2) (March 15, 2021, to March 14, 2022). We chose March 15, 2020, as the onset of the COVID-19 pandemic, aligning with the nationwide emergency declaration for COVID-19 in the United States. Our analysis primarily compares the Pre-Pandemic Year with Pandemic Years 1 and 2.

### Study population

To define a comparable study population over the three study time windows, we applied several exclusion criteria. First, clinicians who consistently provided the minimum level of service throughout the study period were identified. Clinicians who had no visits at least six months, those with fewer than 250 visits (total), or those with more than 12,500 visits in a single calendar year between 2019 and 2022 were excluded from our study database. We selected calendar years for high-volume exclusion because subscriptions to the registry are usually based on them. After these exclusions, our database included 1,651 primary care clinicians from 518 primary care practices across 45 states.

We also excluded approximately 3% of patients due to missing essential demographic (age, sex) or geographic (ZIP code) information. After implementing these criteria, our (repeated cross-sectional) sample comprised 1,338,824 unique patients in the Pre-Pandemic Year, 1,225,047 unique patients in Pandemic Y1, and 1,233,474 unique patients in Pandemic Y2.

Patients were classified into two groups based on their age at the time of each visit: those aged 0–17 years were defined as children/adolescents, and those aged 18 years and older as adults. This categorization aligns with Advisory Committee on Immunization Practices (ACIP) guidelines for age-specific routine immunization schedules. Only patients with at least one visit in each time window were included in the denominator for vaccination rate calculations.

### Outcome measures and other study variables

Our primary outcome measure was to ascertain, whether individual patients seen in the practice received at least one non-COVID-19 vaccination during a primary care office visit in each study time window, as recorded in the PRIME registry. We included only routine vaccinations that were recommended by the ACIP for each age group—children/adolescents and adults—during the study period [24]. Vaccines recommended for special circumstances, such as international travel and COVID-19 vaccinations, which were outside the scope of this study were excluded. Vaccines were identified using the Current Procedural Terminology/Healthcare Common Procedure Coding System codes from the EHR (S1 Table). To examine trends at the practice level, we also calculated the practice-level volume of non-COVID-19 vaccinations and total office visits for each month during the study period.

To account for patient characteristics, our independent variables included sex, race, ethnicity, rurality, and residence in historically marginalized areas. As documented in the EHR, patient race was categorized as white, black, or African American, while ethnicity was categorized as Hispanic or Latino versus non-Hispanic or Latino. The 2010 Rural-Urban Commuting Area Codes (RUCA) developed at the University of Washington were used to classify patient zip codes as urban or rural [25]. These codes classify U.S. census tracts based on population density, urbanization, and commuting patterns. Residence in a historically marginalized area was assessed using the Social Deprivation Index (SDI) of the patient’s county of residence [26]. The SDI is derived from county-level socioeconomic indicators and reflects an individual’s exposure to historical disinvestment in resources and marginalization. A patient’s residence was classified as having a high degree of marginalization if the SDI for the county was higher than 50.

### Statistical analyses

To analyze trends in primary care-based vaccination rates over time, we compared the number of unique individuals across age groups (child-adolescent versus adult) and by study year, using chi-square tests to assess differences in those who did and did not receive a non-COVID-19 vaccine through their primary care practices. Additionally, we assessed monthly practice-level trends by age group. This was done by dividing the number of unique patients in each age group, practice, and month who received at least one non-COVID-19 vaccine by the total number of patients in that age group and practice who had an office visit in the same month. To highlight the impact of the pandemic on seasonal trends in vaccinations, we created rate differences by month to compare Pandemic Years 1 and 2 with our baseline Pre-Pandemic Year. This allowed us to compare the rates for January 2021 (Pandemic Y1) and January 2022 (Pandemic Y2) with those for January 2020 (Pre-Pandemic Year). To examine the impact of the pandemic on the overall volume of vaccinations, as opposed to patients receiving at least one vaccine, we also calculated the volume of vaccine doses administered in each time period.

To assess the association between independent variables and vaccinated individuals in each period, we developed two clustered multivariable logistic regression models. Model 1 included only demographic variables, whereas binary age groups were included as control variables in Model 2. The models were clustered at the practice level to account for the unmeasured confounders.

Statistical analyses were performed using Stata 17.0 (StataCorp, College Station, TX, USA). Statistical significance was set at a two-sided p-value <0.05. This study was approved by the American Academy of Family Physicians (AAFP) Institutional Review Board (IRB Application #22–450; Approval Date: 08/30/2022; Amendment #1 Approval Date: 11/02/2022). The IRB waived the requirement for informed consent as the study involved secondary analysis of de-identified electronic health record data. No identifiable private information was accessed by the researchers.

## Results

**Fig 1:**
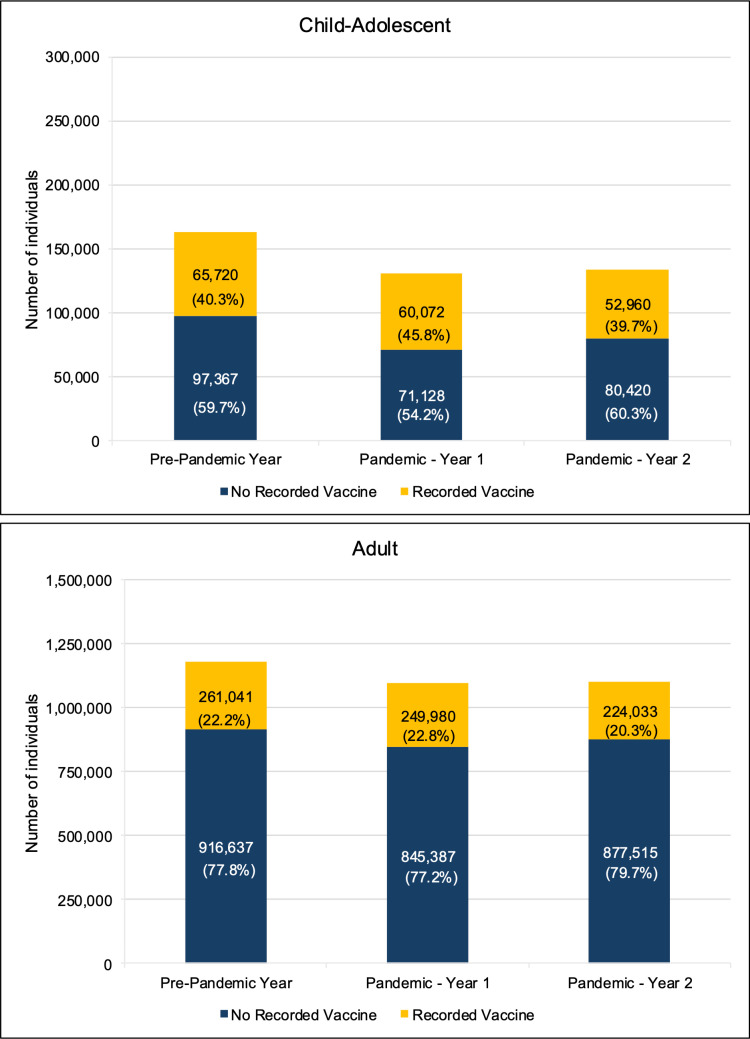
Vaccination status of child-adolescent and adult individuals across pre-pandemic and pandemic years.

Fig 1 shows the number of vaccinated individuals, categorized by age group, across three distinct periods. The number of vaccinated individuals decreased by 9.6% among children-adolescents and 4.2% among adults in Pandemic Year 1. This decline became more pronounced in Pandemic Year 2, with a 19.4% decrease among children-adolescents and a 14.2% decrease among adults compared to the Pre-Pandemic Year.

Table 1 indicates the distribution of study population characteristics by vaccination status. In the pre-pandemic year, 49.7% of vaccinated child-adolescent individuals were female, 63.0% were white, 67.5% of them resided in urban areas and 52.9% had an SDI < 51. For adults, 56.1% were female, 81.2% were white, 77.8% of them resided in urban areas and 55.2% had an SDI < 51. In Pandemic Y1, 49.6% of vaccinated child-adolescent individuals were female, 61.2% were white, 67.5% of them resided in urban areas and 51.6% had an SDI < 51. For adults, 55.8% were female, 81.2% were white, 79.1% of them resided in urban areas, and 56.2% had an SDI < 51. In Pandemic Y2, 49.6% of vaccinated child-adolescent individuals were female, 58.3% were white, 68.1% of them resided in urban areas, and 50.6% had an SDI < 51. For adults, 55.8% were female, 80.2% were white, 79.4% of them resided in urban areas, and 55.5% had an SDI < 51. Vaccination status comparisons for each variable, within each age group and study period, were statistically significant (p < 0.001).

**Table 1:** Distribution of study population characteristics by vaccination status in each study period.

	Pre-Pandemic Year				Pandemic – Year 1				Pandemic – Year 2			
	Child-Adolescent		Adult		Child-Adolescent		Adult		Child-Adolescent		Adult	
	n (%)		n (%)		n (%)		n (%)		n (%)		n (%)	
	Vaccinated	Total	Vaccinated	Total	Vaccinated	Total	Vaccinated	Total	Vaccinated	Total	Vaccinated	Total
**Sex** ^a^												
Female	32,636 (49.7)	81,746 (50.2)	146,348 (56.1)	665,004 (56.5)	29,804 (49.6)	66,054 (50.4)	139,284 (55.8)	616,471 (56.4)	26,256 (49.6)	66,873 (50.1)	124,988 (55.8)	619,935 (56.3)
Male	33,011 (50.3)	81,100 (49.8)	114,460 (43.9)	511,510 (43.5)	30,231 (50.4)	64,964 (49.6)	110,480 (44.2)	477,160 (43.6)	26,700 (50.4)	66,474 (49.9)	98,920 (44.2)	481,295 (43.7)
Total	65,647 (100.0)	162,846 (100.0)	260,808 (100.0)	1,176,514 (100.0)	60,035 (100.0)	131,018 (100.0)	249,764 (100.0)	1,093,931 (100.0)	52,956 (100.0)	133,347 (100.0)	223,908 (100.0)	1,101,230 (100.0)
**Race/Ethnicity** ^a^												
White	35,352 (63.0)	92,599 (68.9)	190,971 (81.2)	806,781 (79.9)	32,159 (61.2)	74,305 (67.2)	184,274 (81.2)	753,345 (79.8)	26,440 (58.3)	71,665 (66.0)	161,140 (80.2)	738,795 (79.5)
Hispanic or Latino	13,985 (24.9)	26,410 (19.7)	21,312 (9.1)	87,239 (8.6)	13,874 (26.4)	23,145 (20.9)	20,144 (8.9)	80,804 (8.6)	12,947 (28.5)	23,683 (21.8)	18,310 (9.1)	79,541 (8.6)
Black or African American	5,053 (9.0)	11,267 (8.4)	16,487 (7.0)	88,115 (8.7)	4,804 (9.1)	9,558 (8.6)	16,317 (7.2)	84,686 (9.0)	4,418 (9.7)	9,579 (8.8)	15,572 (7.8)	84,849 (9.1)
Other	1,740 (3.1)	4,041 (3.0)	6,404 (2.7)	28,226 (2.8)	1,732 (3.3)	3,569 (3.2)	6,234 (2.7)	25,102 (2.7)	1,554 (3.4)	3,705 (3.4)	5,846 (2.9)	2,635 (0.3)
Total	56,130 (100.0)	134,317 (100.0)	235,174 (100.0)	1,010,361 (100.0)	52,569 (100.0)	110,577 (100.0)	226,969 (100.0)	943,937 (100.0)	45,359 (100.0)	108,632 (100.0)	200,868 (100.0)	929,535 (100.0)
**RUCA** ^a^												
Urban	43,996 (67.5)	94,755 (58.6)	202,178 (77.8)	859,859 (73.4)	40,377 (67.5)	76,500 (58.6)	196,821 (79.1)	798,468 (73.3)	35,776 (68.1)	76,559 (57.8)	177,056 (79.4)	798,945 (72.9)
Rural	21,217 (32.5)	67,035 (41.4)	57,588 (22.2)	31,119 (22.7)	19,415 (32.5)	54.010 (41.4)	51,882 (20.9)	291,274 (26.7)	16,769 (31.9)	55,876 (42.2)	45,875 (20.6)	297,183 (27.1)
Total	65,213 (100.0)	161,790 (100.0)	259,766 (100.0)	1,171,049 (100.0)	59,792 (100.0)	130,510 (100.0)	248,703 (100.0)	1,089,742 (100.0)	52,545 (100.0)	132,435 (100.0)	222,931 (100.0)	1,096,128 (100.0)
**SDI** ^a^												
0-50	34,336 (52.9)	79,121 (48.6)	143,407 (55.2)	588,613 (51.6)	30,839 (51.6)	62,457 (47.1)	139,690 (56.2)	548,394 (51.3)	26,570 (50.6)	63,219 (46.4)	123,632 (55.5)	553,340 (51.0)
51-100	30,878 (47.5)	83,629 (51.4)	116,358 (44.8)	551,145 (48.4)	28,956 (48.4)	70,157 (52.9)	109,014 (43.8)	520,368 (48.7)	25,977 (49.4)	73,130 (53.6)	99,297 (44.5)	531,260 (49.0)
Total	64,944 (100.0)	162,750 (100.0)	259,765 (100.0)	1,139,758 (100.0)	59,795 (100.0)	132,614 (100.0)	248,704 (100.0)	1,068,762 (100.0)	52,547 (100.0)	136,349 (100.0)	222,929 (100.0)	1,084,600 (100.0)

^a^p < 0.001; SDI: Social Deprivation Index; RUCA: Rural-Urban Commuting Area Codes.

Compared to that of Pre-Pandemic Year, there was a decline in the total number of vaccine doses administered during the pandemic (S2 Table). Among children and adolescents, the administered vaccine doses decreased by 9.6% in Pandemic Y1 and 14.9% in Pandemic Y2. For adults, a similar trend was observed, with decreases of 4.7% and 15.3%, respectively, during the same period.

**Fig 2:**
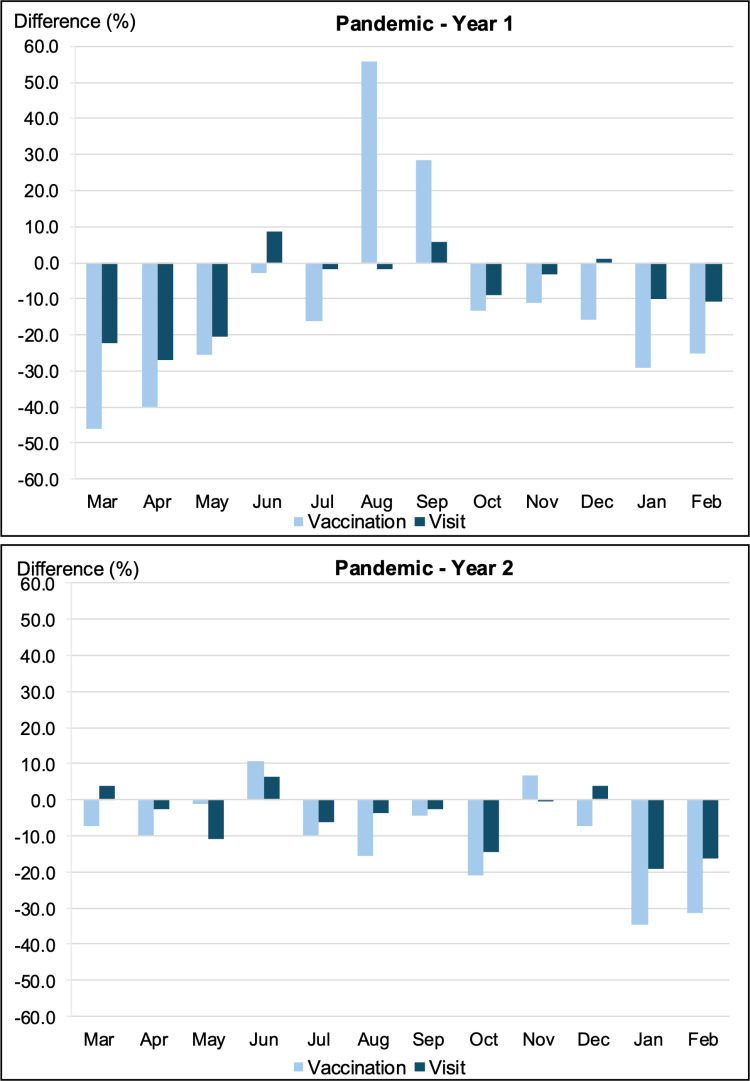
Changes in vaccination and visit rates during pandemic years compared with the pre-pandemic period.

Fig 2 compares the differences in vaccination and visit rates for all patients between the two Pandemic Years and the Pre-Pandemic Year. Overall, the monthly visit and vaccination rates declined during the pandemic compared to the those of the Pre-Pandemic Year, with a few exceptions. During Pandemic Y1, both visit and vaccination rates improved only in certain months (visits: June, September and December; vaccinations: August, and September) compared to those the Pre-Pandemic Year. Throughout Pandemic Y2, visits exceeded pre-pandemic levels in just three months (March, June, and December), whereas vaccination rates remained consistently lower, except in June and November. Despite a partial recovery in visit rates during Pandemic Y2, vaccination rates continued to be low. With the exception of August and September in the 1st year and May, June and November in the 2nd year, differences in vaccination rates were worse than visit rates during the remaining months of the pandemic. While the monthly trends in vaccination and visit rates were similar among adults compared to overall sample, except May and July in the 2nd year, the child-adolescent group had different trends in both years (S1 and S2 Figs). In both years, visit rates decreased more than vaccination rates in four months (April, May, October and February in the 1st year and March, May, June and February in the 2nd year).

In logistic regression models, as presented in Model 1, females, rural residents, and individuals living in areas with higher (SDI > 50) social risk had the lowest odds of vaccination in all three time periods (p < 0.05 in each period) (Table 2). This pattern persisted even after introducing age adjustment in Model 2, where females, rural residents and individuals living in areas with higher (SDI > 50) social risk maintained the lowest odds of vaccination before and during the pandemic (p < 0.05). As the pandemic continued, individuals living in rural areas had lower odds of being vaccinated in both models, whereas the odds remained similar within groups with higher social risk.

**Table 2:** Logistic regression models estimating the odds ratio for vaccination in each period.

	Pre-Pandemic Year Odds ratio (95% CI)	Pandemic Year 1 Odds ratio (95% CI)	Pandemic Year 2 Odds ratio (95% CI)
**Sex**			
Female	Ref	Ref	Ref
Male	1.15 (1.11–1.20)	1.12 (1.08–1.16)	1.16 (1.12–1.21)
**Race/Ethnicity**			
White	Ref	Ref	Ref
Hispanic or Latino	1.63 (1.23–2.16)	1.28 (1.01–1.63)	1.69 (1.24–2.29)
Black or African-American	0.82 (0.68–0.99)	0.77 (0.66–0.89)	0.86 (0.71–1.05)
Other	1.16 (0.91–1.49)	1.15 (0.90–1.47)	1.24 (0.96–1.62)
**RUCA**			
Urban	Ref	Ref	Ref
Rural	0.72 (0.58–0.88)	0.67 (0.54–0.84)	0.63 (0.51–0.79)
**SDI**			
0–50	Ref	Ref	Ref
51–100	0.72 (0.56–0.92)	0.70 (0.54–0.90)	0.73 (0.57–0.94)
Age-Adjusted			
**Sex** ^**a**^			
Female	Ref	Ref	Ref
Male	1.10 (1.06–1.14)	1.12 (1.08–1.16)	1.11 (1.07–1.16)
**Race/Ethnicity**			
White	Ref	Ref	Ref
Hispanic or Latino	1.31 (1.03–1.67)	1.28 (1.01–1.63)	1.31 (1.00–1.71)
Black or African-American	0.80 (0.69–0.92)	0.77 (0.54–0.88)	0.84 (0.72–0.98)
Other	1.10 (0.87–1.40)	1.15 (0.89–1.47)	1.16 (0.90–1.49)
**RUCA**			
Urban	Ref	Ref	Ref
Rural	0.61 (0.49–0.76)	0.56 (0.45–0.70)	0.54 (0.43–0.67)
**SDI**			
0–50	Ref	Ref	Ref
51–100	0.72 (0.57–0.91)	0.69 (0.54–0.88)	0.72 (0.57–0.93)

SDI: Social Deprivation Index; RUCA: Rural-Urban Commuting Area Codes.

## Discussion

Receipt of non-COVID-19 vaccines through primary care declined significantly during the pandemic. These declines were consistent across age groups—children, adolescents, and adults—and persisted over time. This pattern aligns with prior studies showing substantial disruptions in routine immunizations during the early stages of the COVID-19 pandemic, as in-person care was postponed or avoided [6–9]. Although some recoveries were noted, it was inconsistent, with disparities persisting among vulnerable groups. Some studies showed that childhood vaccination rates remained below pre-pandemic levels into 2021, particularly among lower-income and racially or ethnically minoritized populations [27–30]. Our study contributes to this literature by focusing specifically on primary care settings and by examining vaccination trends across rural and socioeconomically disadvantaged populations, highlighting the potential for lasting declines in vaccine uptake in these communities.

Primary care visits also declined during the pandemic, across all age groups, with a partial recovery in Pandemic Year 2. A study examining COVID-19 vaccinations from the same database for 2021 also showed a decline in patient visits, which reduced the opportunity for vaccination [30]. In a nationwide study similar to ours, visit rates improved faster than vaccination rates across all age groups [11]. Although reduced in-person care and the rise of telehealth may have contributed to missed vaccinations [31], our results suggest that reduced access alone does not fully explain the sustained drop in vaccination rates.

A notable exception to the overall trend was a sharp spike in vaccinations observed in August 2020, likely reflecting a seasonal surge in back-to-school immunizations. Many schools and local authorities continued to enforce vaccine requirements despite the pandemic, prompting families to seek care during this period. Eased restrictions in some areas may have also contributed to a temporary increase in in-person visits. However, this rebound was short-lived and did not lead to sustained recovery, especially among adults and patients in rural or socioeconomically deprived areas.

Our analysis of patient characteristics highlighted trends consistent with earlier literature: rural residents, and those living in areas of high social deprivation were less likely to receive vaccines across all time periods [7,9]. Rural residents, however, appear particularly at risk for low vaccination rates after the onset of the pandemic. We found that individuals living in rural areas had even lower odds of vaccine receipt in Pandemic Year 2 compared with Pandemic Year 1. These findings underscore the need for targeted outreach and support for rural populations, where structural barriers and limited access to care may compound pandemic-related disruptions.

While these trends suggest meaningful gaps in vaccine delivery, changes in the composition of patients visiting primary care may also help explain the observed trends. For instance, individuals who sought care during the pandemic may differ in age, health status, or vaccine eligibility from those who delayed care [32,33]. Shifts in visit type—from preventive to acute—could also reduce opportunities for vaccination. However, the persistence of declines across subgroups and time periods supports the interpretation that structural disruptions in care delivery were a key driver.

Our findings have several implications for clinical practice and public health. Catch-up vaccination efforts are urgently needed, particularly in rural and socioeconomically disadvantaged communities. Strategies could include mobile clinics, school-based drives, and reminder systems. At the policy level, stronger support is needed to help primary care practices sustain essential preventive services during public health crises. Enhancing standardized reporting tools, reminders, and patient outreach systems could help clinicians more effectively identify and reach under-vaccinated individuals. These efforts are critical to ensuring continuity of care and preventing widening immunization gaps during future disruptions.

Our study contributes to a growing body of literature on the impact of the COVID-19 pandemic on overall vaccination rates. Similar to other studies, our results suggest consistent declines of vaccinations across all age groups. Particular strengths of our study are the use of a national sample of registry data (claims and electronic health records) from primary care settings, a full two years’ follow-up data to onset of the pandemic, and detailed patient characteristics to identify vulnerable subgroups with a modest proportion of rural individuals each year (20–35%).

A number of study limitations should be noted. First, primary care participation in the PRIME registry is voluntary and not necessarily representative of all primary care practices across the country. Second, PRIME data capture the encounters for participating practices, rather than a consistent set of patients over time. Thus, our study data contain repeated cross-sectional information rather than a longitudinal cohort of the same individuals over time and an individual might be unique within each time period. Finally, it is important to note that PRIME data only capture care provided by primary care practices, we cannot rule out the possibility that individuals may have shifted their site of care for receipt of vaccinations in response to the pandemic.

## Conclusions

Our study provides further evidence that the COVID-19 pandemic was associated with declines in vaccination rates. It is noteworthy that despite the improvement in visits, the decline in vaccination rates persisted into the second year of the pandemic. These declines will increase risk of outbreaks of vaccine-preventable diseases, resurgence of diseases that were previously under control or eliminated, and additional health disparities for immunocompromised individuals. We also find evidence that individuals in rural areas may be particularly at risk for declining vaccination rates, highlighting an important target population for new programs and interventions.

## Supporting information

S1 Table Included vaccines with CPT codes. (PDF)

S2 Table Total number of vaccination doses during the study period. (PDF)

S1 Fig Changes in vaccination and visit rates among adult group during pandemic years compared with the pre-pandemic period. (TIF)

S2 Fig Changes in vaccination and visit rates among child-adolescent group during pandemic years compared with the pre-pandemic period. (TIF)
