# Supplementary material for: The impact of the COVID-19 pandemic on vaccinations in United States primary care practices
Source: PLoS One. 2025 Jun 10;20(6):e0325934. doi: 10.1371/journal.pone.0325934 (PMC12151362; doi:10.1371/journal.pone.0325934)
Supplement: S1 Table — (PDF) [file pone.0325934.s001.pdf]

S1 Table: Included vaccines with CPT codes

Vaccines	Child-Adolescent	Adult
Hepatitis B	90636, 90697, 90723, 90739, 90743, 90744, 90745, 90747, 90759	90636, 90739, 90746
Rotavirus	90680, 90681	-
Diphtheria, tetanus, & acellular pertussis	90696, 90697, 90698, 90700, 90723	-
Haemophilus influenzae type b	90647, 90648, 90697, 90698	-
Pneumococcal vaccine	90670, 90671	90671, 90677, 90732
Inactivated poliovirus	90696, 90697, 90698, 90713, 90723	-
Measles, mumps, rubella	90707, 90710	-
Varicella	90710, 90716	-
Hepatitis A	90633, 90634, 90636	-
Tetanus, diphtheria, & acellular pertussis	90715	90715
Meningococcal ACWY	90619, 90733, 90734	-
Human papillomavirus	90649, 90650, 90651	90649, 90650, 90651
Zoster recombinant	-	90736, 90750
Influenza	90470, 90630, 90653, 90654, 90655, 90656, 90657, 90658, 90659, 90660, 90661, 90662, 90663, 90664, 90666, 90668, 90672, 90674, 90685, 90686, 90687, 90688, 90694, 90756	90470, 90630, 90653, 90654, 90655, 90656, 90657, 90658, 90659, 90660, 90661, 90662, 90663, 90664, 90666, 90668, 90672, 90673, 90674, 90682, 90685, 90686, 90687, 90688, 90694, 90724, 90756
