# Supplementary material for: The impact of the COVID-19 pandemic on vaccinations in United States primary care practices
Source: PLoS One. 2025 Jun 10;20(6):e0325934. doi: 10.1371/journal.pone.0325934 (PMC12151362; doi:10.1371/journal.pone.0325934)
Supplement: S2 Table — (PDF) [file pone.0325934.s002.pdf]

S2 Table: Total number of vaccination doses during the study period

Vaccines	Pre-Pandemic Year	Pandemic – Year 1	Pandemic – Year 2
**Child-Adolescent**			
Influenza	32,512	30,868	21,341
Human papillomavirus	12,701	10,365	9,593
Hepatitis A	12,454	10,640	10,272
Meningococcal ACWY	13,775	12,333	12,079
Varicella	12,487	11,002	11,035
Tetanus, diphtheria, & acellular pertussis	7,771	7,345	6,946
Measles, mumps, rubella	12,165	10,944	11,056
Hepatitis B	9,636	8,634	8,769
Inactivated poliovirus	16,956	15,117	15,467
Diphtheria, tetanus, & acellular pertussis	21,253	19,481	19,683
Rotavirus	6,861	6,397	6,402
Pneumococcal vaccine	12,055	11,353	11,263
Haemophilus influenzae type b	13,924	12,901	13,017
**Total**	184,550	167,380	156,923
**Adult**			
Hepatitis B	3,579	1,967	1,841
Pneumococcal vaccine	26,104	23,331	18,367
Tetanus, diphtheria, & acellular pertussis	40,386	29,679	28,503
Human papillomavirus	2,288	1,659	1,637
Influenza	242,327	234,770	203,875
Zoster	13,808	17,905	17,829
**Total**	328,492	309,311	272,052
