# Supplementary figures and images for: The impact of the COVID-19 pandemic on vaccinations in United States primary care practices
Source: PLoS One. 2025 Jun 10;20(6):e0325934. doi: 10.1371/journal.pone.0325934 (PMC12151362; doi:10.1371/journal.pone.0325934)

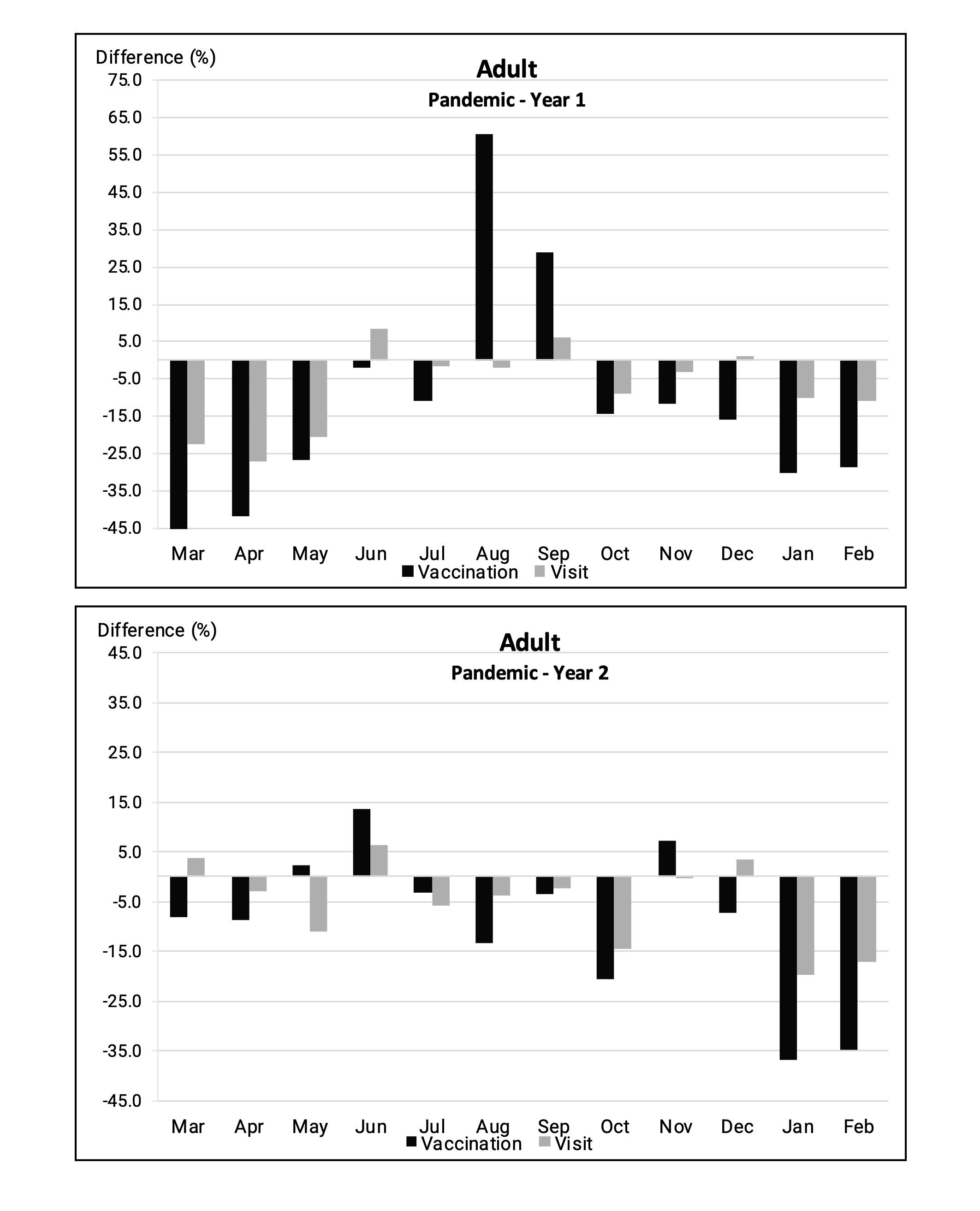

Supplement: S1 Fig — (TIF) [file pone.0325934.s003.tif]

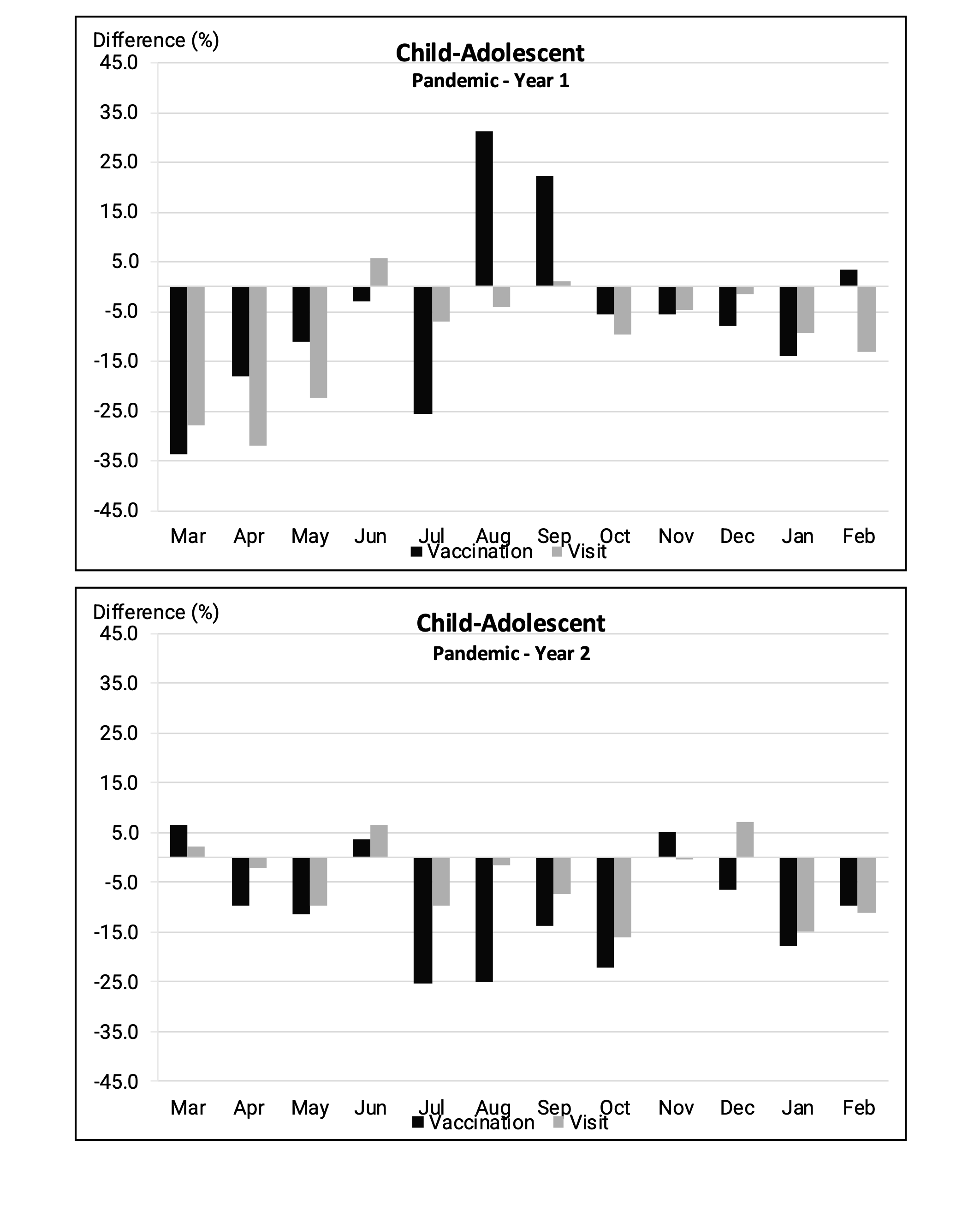

Supplement: S2 Fig — (TIF) [file pone.0325934.s004.tif]
